# Clinical characteristics and mortality risk factors among hospitalized infants and children with pertussis in China: a retrospective study

**DOI:** 10.3389/fpubh.2025.1663885

**Published:** 2025-11-17

**Authors:** Shuhan Zheng, Feng Luo, Ying Su, Xinyu Liu, Rongrong Dai, Xuan Deng, Yao Zhu, Yang Zhou, Hanqing He, Chunzhen Hua, Hangjie Zhang

**Affiliations:** 1 School of Public Health, Hangzhou Medical College, Hangzhou, China; 2 School of Public Health, Zhejiang Chinese Medical University, Hangzhou, China; 3 Center for General Practice Medicine,Department of Research Administration Dept.Zhejiang Provincial People’s Hospital(Affiliated People’s Hospital), Hangzhou Medical College, Hangzhou, Zhejiang, China; 4 Department of Immunization Program, Zhejiang Provincial Center for Disease Control and Prevention, Hangzhou, China; 5 Zhejiang Key Lab of Vaccine, Infectious Disease Prevention and Control, Zhejiang Provincial Center for Disease Control and Prevention, Hangzhou, China; 6 Department of Infectious Diseases, National Clinical Research Center for Child Health, The Children’s Hospital, Zhejiang University School of Medicine, Hangzhou, China; 7 Department of Prevention and Control of Infectious Disease, Zhejiang Provincial Center for Disease Control and Prevention, Hangzhou, China

**Keywords:** pertussis, clinical characteristic, pediatric intensive care unit, infants and children, mortality risk factor

## Abstract

**Background:**

The resurgence of pertussis has emerged as a significant global public health threat. Severe pertussis primarily affects infants and children and is often associated with serious complications or death.

**Methods:**

We retrospectively evaluated the clinical data of 197 hospitalized children with pertussis admitted to Children’s Hospital, Zhejiang University School of Medicine, from 2019 to 2024. Patients were categorized into severe and mild cases groups based on their admission ward. Multivariate logistic model was applied to explore associated factors.

**Results:**

Among the 197 hospitalized children, those born prematurely or younger than 3 months were more likely to be admitted to the pediatric intensive care unit (PICU). All patients admitted to the PICU were infants who were either unvaccinated or incompletely vaccinated against pertussis. The most common clinical symptoms among hospitalized children were pneumonia, paroxysmal cough, and flushing. The severe cases group exhibited significantly higher levels than the mild cases group in length of stay, duration of antibiotic treatment, white blood cell (WBC) count, pneumonia, respiratory failure, and tachypnea (*p* < 0.05). Multivariate logistic regression analysis identified cefuroxime usage [odds ratio (OR) = 0.10, 95% confidence interval (CI) = 0.01–0.92], high WBC count (OR = 0.79, 95% CI = 0.69–0.91), and high neutrophil percentage (OR = 1.49, 95% CI = 1.22–1.82) as independent risk factors for PICU admission (*p* < 0.05).

**Conclusion:**

Infants younger than 3 months and children who are unvaccinated or incompletely vaccinated for pertussis are more susceptible to developing severe pertussis. Common clinical features of severe pertussis include pneumonia, respiratory failure, elevated WBC count, and decreased neutrophil percentage. Pediatricians should prioritize early recognition and treatment of these clinical indicators to prevent the progression of severe pertussis.

## 1 Introduction

Pertussis is an acute respiratory infectious disease caused by *Bordetella pertussis*. It is transmitted through respiratory droplets and affects individuals of all age groups worldwide (1). The primary clinical feature of pertussis is a paroxysmal cough. Clinical manifestations vary depending on factors such as the patient’s age, vaccination status, and antibiotic usage (2).

Since the 1980s, several countries with high vaccine coverage have experienced a “pertussis resurgence,” marked by an increase in the incidence of pertussis after years of maintaining low levels, including outbreaks (3). Notably, the emergence of the COVID-19 virus and the subsequent prevention and control measures have significantly altered the epidemiological trajectory of pertussis. During the COVID-19 pandemic (2020–2021), strict non-pharmaceutical interventions (mask-wearing, maintaining social distancing) implemented worldwide effectively controlled COVID-19. Concurrently, they unexpectedly drove the incidence of other respiratory infectious diseases, including pertussis, to a historic low (4). However, this suppression of transmission also resulted in the accumulation of pertussis susceptibility among vulnerable populations, particularly infants and children, a phenomenon referred to as “immune debt.” Consequently, a strong resurgence of pertussis outbreaks has been observed across many regions globally since 2023 (5). According to the World Health Organization’s 2023 pertussis report, 159,832 pertussis cases were reported globally, a figure 5.25 times higher than the 30,402 cases reported in 2021 (6).

The rising incidence of pertussis has become a significant global public health threat. In China, the incidence of pertussis has also been increasing, with rates ranging from 0.31 to 2.88 per 100,000 population between 2020 and 2023 (6). It is projected that the pertussis epidemic will persist at high levels or exhibit periodic peaks in the coming years (7). Furthermore, insufficient awareness of the need to seek medical attention, along with other factors causing delayed or missed diagnoses in patients with atypical pertussis symptoms, suggests that the actual incidence of pertussis may be underestimated (8).

If pertussis is not promptly diagnosed or is misdiagnosed in its early stages, it can easily progress to severe pertussis, which is associated with a higher case-fatality rate. Severe pertussis primarily affects infants and young children and is often accompanied by severe complications such as respiratory failure, encephalopathy, and pulmonary hypertension—significant causes of mortality in this age group (9). According to a global pertussis disease burden model, in 2017, there were 85,900 pertussis-related deaths among children under 1 year old globally, accounting for 53% of deaths in children under 5 years of age (10). Identifying the risk factors for severe pertussis and examining the clinical characteristics of patients with varying disease severity are essential for improving the prognosis of severe pertussis.

This study retrospectively analyzed children with pertussis admitted to the pediatric intensive care unit (PICU) and general ward of Children’s Hospital, Zhejiang University School of Medicine, from 2019 to 2024 (up to May). The aim was to clarify the clinical characteristics of pertussis and the risk factors for severe pertussis, enhance the early identification of severe cases, improve the prognosis, reduce the mortality risk, and provide a reference for accurately monitoring and assessing the impact of pertussis on children’s health. Additionally, the findings aim to support future research on definitive and effective interventions.

## 2 Methods

### 2.1 Study participants

This study retrospectively analyzed pediatric patients with confirmed pertussis who were admitted to the infectious disease department and PICU of Children’s Hospital, Zhejiang University School of Medicine, from February 2019 to May 2024. Hospitalized children with pertussis were categorized into a severe cases group and a mild cases group based on the PICU admission status. In total, 197 children diagnosed with pertussis were included in the study (38 children in the severe cases group matched with 159 children in the mild cases group for comparison). The inclusion criteria were an age of <18 years, regardless of sex; clinical diagnosis of pertussis; and availability of complete clinical and laboratory data. The exclusion criteria were incomplete clinical and laboratory data, refusal of participation by the patient’s parents, and fatal outcomes.

### 2.2 Diagnostic criteria for pertussis

According to the Guidelines for the Diagnosis, Management and Prevention of Pertussis of China (2024 Edition) (11), mild and severe cases of pertussis were defined using the following criteria: (1) Mild cases: Persistent cough lasting at least 2 weeks, with one of the following symptoms: paroxysmal cough (The presence of continuous, uncontrollable paroxysmal coughing that persists for ≥7 days), whooping (Clinical diagnosis was made by senior physicians, with decisions based on objective findings from physical examinations), or post-tussive vomiting. Concurrently, either laboratory isolation of *Bordetella pertussis* or positive results from multiplex polymerase chain reaction (PCR) for *Bordetella pertussis*. (2) Severe cases: Currently, there is no universally accepted diagnostic criterion for severe pertussis in infants and children. According to a study by Yao (12), severe pertussis can be diagnosed in patients presenting with any of the following symptoms: lethargy, inability to eat normally, or a significant reduction in appetite; frequent cyanosis or asphyxia, convulsions, or suspected pertussis encephalopathy; persistent tachypnea (Respiratory rate exceeding the upper limit of age-appropriate normal values during quiet state: <2 months of age, >60 breaths per minute; 2–12 months of age, >50 breaths per minute), with a respiratory rate exceeding 70 breaths per minute and a heart rate exceeding 180 beats per minute; frequent or persistent hypoxemia; or a white blood cell (WBC) count exceeding 30 × 109/L, accompanied by pulmonary hypertension. In this study, patients meeting the clinical diagnostic criteria for pertussis and admitted to the PICU were defined as the severe pertussis.

### 2.3 Clinical data collection

In investigating the risk factors for severe pertussis in infants and children, data were collected for both the severe and mild cases groups. Case data of hospitalized children were collected via the inpatient electronic medical record system. The collected data included demographic information, such as patient sex, preterm birth status, age at onset, history of pertussis vaccination, and contact with suspected cases. Laboratory data included routine blood examination results and pertussis nucleic acid test results, all samples were collected within 24 h of the pediatric patients’ hospital admission. Cytokine concentrations were measured using EDTA-anticoagulated plasma samples. After centrifugation and aliquoting, the samples were stored at −80 °C until batch analysis. A commercial human cytokine multiplex assay kit (based on Luminex xMAP technology) was used for detection, with strict adherence to the manufacturer’s standard operating protocols. Complete blood count (CBC) analysis was conducted using an automated hematology analyzer (Sysmex XN-Series). Clinical data included complications, clinical presentations, length of hospital stay, and antibiotic usage status.

### 2.4 Statistical analysis

Statistical analyses were performed using SPSS 24.0 and R 4.3.2 software. Measurement data with a normal or approximately normal distribution are expressed as mean ± standard deviation, and comparisons between groups were conducted using the independent two-sample *t*-test. Measurement data with a skewed distribution are expressed as median and interquartile range, and comparisons between groups were conducted using non-parametric tests. Enumeration data are presented as frequency or percentage, and comparisons between groups were performed using the chi-square test or Fisher’s exact probability test. Variables identified through univariate analysis were included in a multivariate logistic regression to assess the risk factors for admission of patients with pertussis to the PICU. A difference was considered statistically significant at a *p*-value of <0.05.

## 3 Results

### 3.1 Comparison of demographic data

The distribution of the severe and mild cases groups by sex, prematurity, age, vaccination status, and contact history with suspected cases is shown in Table 1.

**Table 1:** Demographic and baseline characteristics of 197 hospitalized children with pertussis.

Characteristics	Total (*n* = 197)	Mild cases (*n* = 159)	Severe cases (*n* = 38)	*χ*^2^/Fisher	*p*
Gender, *n* (%)				0.73	0.392
Male	107 (54.31)	84 (78.50)	23 (21.50)		
Female	90 (45.69)	75 (83.33)	15 (16.67)		
Prematurity, *n* (%)				8.14	0.004
Term birth	165 (83.76)	139 (84.24)	26 (15.76)		
Preterm birth	32 (16.24)	20 (62.50)	12 (37.50)		
Age groups, *n* (%)				-	<0.001
<3 months	55 (27.92)	42 (76.36)	13 (23.64)		
3–12 months	85 (43.15)	62 (72.94)	23 (27.06)		
1–5 years	12 (6.09)	10 (83.33)	2 (16.67)		
>5 years	45 (22.84)	45 (100.00)	0 (0.00)		
Vaccination status, *n* (%)				32.73	<0.001
Not vaccinated	72 (36.55)	45 (62.50)	27 (37.50)		
Not fully vaccinated	73 (37.05)	62 (84.93)	11 (15.07)		
Fully vaccinated	52 (26.40)	52 (100.00)	0 (0.00)		
Suspected cases contact history					
Parents, *n* (%)				0.23	0.632
Yes	56 (28.43)	44 (78.57)	12 (21.43)		
No	141 (71.57)	115 (81.56)	26 (18.44)		
Siblings, *n* (%)				3.44	0.064
Yes	68 (34.52)	50 (73.53)	18 (26.47)		
No	129 (65.48)	109 (84.50)	20 (15.50)		
Grandparents, *n* (%)				0.06	0.806
Yes	23 (11.68)	19 (82.61)	4 (17.39)		
No	174 (88.32)	140 (80.46)	34 (19.54)		

*χ*^2^, chi-square test; -: Fisher’s exact test.

A total of 197 children diagnosed with pertussis were included in this study, with 38 patients admitted to the PICU. Among the hospitalized patients, 107 were male (54.31%) and 90 were female (45.69%). There were 165 term infants (83.76%) and 32 preterm infants (16.24%). Regarding the age at onset, 55 patients (27.92%) were younger than 3 months, 85 (43.15%) were between 3 and 12 months, 12 (6.09%) were between 1 and 5 years, and 45 (22.84%) were over 5 years. Premature infants and those younger than 3 months were more likely to be admitted to the PICU, with statistically significant differences (*p* < 0.05). However, no statistically significant difference in the sex distribution was observed between the two groups (*p* > 0.05).

Among the 197 patients in this study, 52 (26.40%) had completed the full series of pertussis vaccination, while 72 (36.55%) were unvaccinated and 73 (37.05%) were partially vaccinated. All patients admitted to the PICU were either unvaccinated or had not completed the full series of pertussis vaccination, with a statistically significant difference in vaccination history between the two groups (*p* < 0.05). Regarding contact with suspected cases, 56 (28.43%) patients had contact with parents, 68 (34.52%) with siblings, and 23 (11.68%) with grandparents. However, there was no statistically significant difference in contact with suspected cases between the two groups (*p* > 0.05).

### 3.2 Comparison of laboratory data

**Figure 1:**
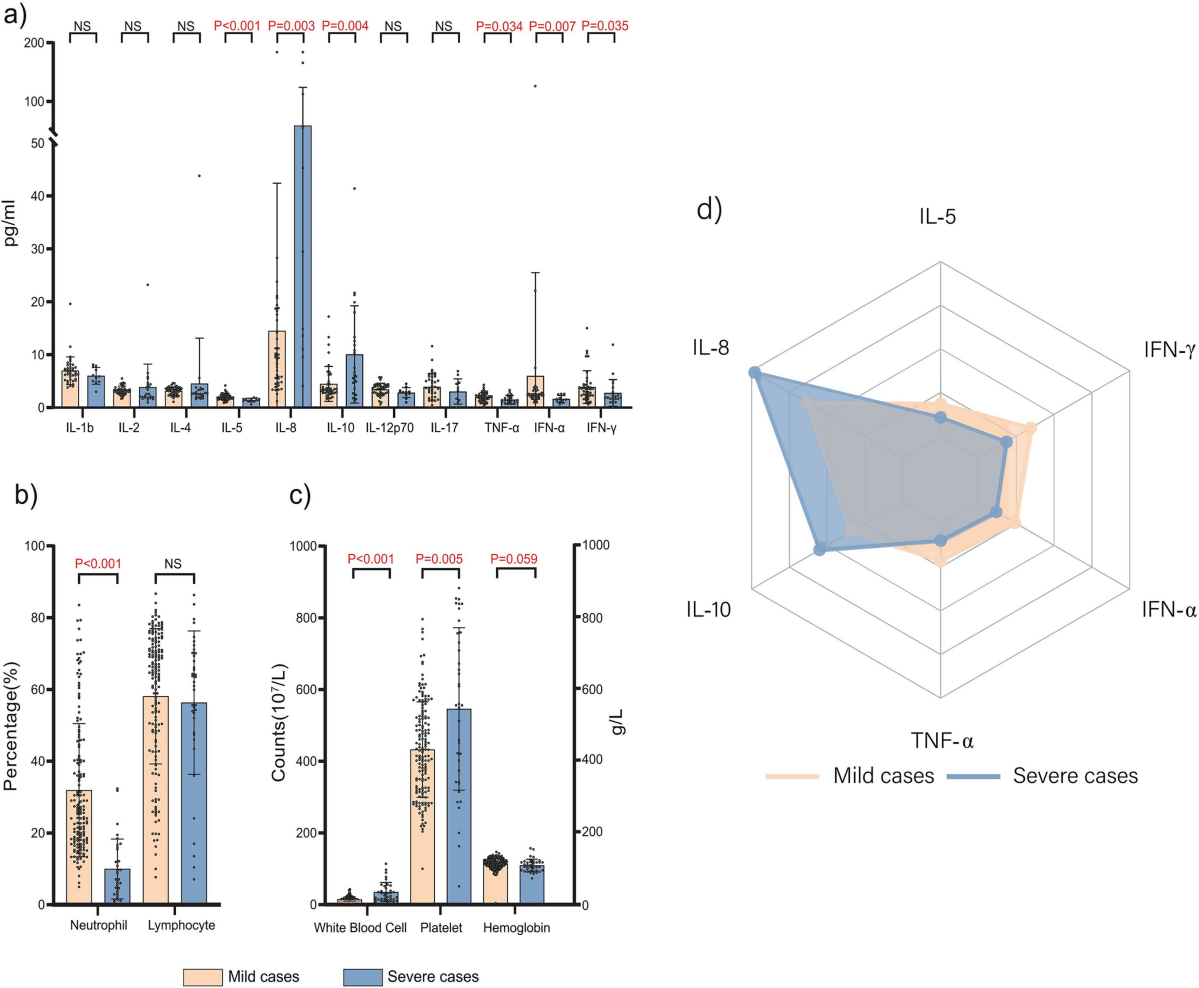
Laboratory characteristics of 197 hospitalized children with pertussis.

The distribution of laboratory characteristics between the severe and mild cases groups is illustrated in Figure 1.

In this study, 115 patients (58.37%) showed varying degrees of elevated WBC counts, with 24 (12.18%) having a WBC of >30 × 109/L. Comparisons of laboratory data between the two groups revealed that the severe cases group had significantly higher interleukin (IL)-8 and IL-10 levels (Figures 1a–1d), WBC counts, and platelet counts (Figure 1c) than the mild cases group (*p* < 0.05). Conversely, the severe cases group exhibited significantly lower levels of IL-5, tumor necrosis factor (TNF)-*α*, interferon (IFN)-α, IFN-*γ*, and neutrophil percentage (Figure 1b), than the mild cases group (*p* < 0.05).

### 3.3 Comparison of clinical data

The distribution of complications and clinical presentations between the severe and mild cases groups is detailed in Tables 2, 3.

**Table 2:** Complications and clinical presentations of 197 hospitalized children with pertussis.

Characteristics	Total (*n* = 197)	Mild cases (*n* = 159)	Severe cases (*n* = 38)	*χ*^2^/Fisher	*p*
Pneumonia, *n* (%)				16.13	<0.001
No	77 (39.09)	73 (94.81)	4 (5.19)		
Yes	120 (60.91)	86 (71.67)	34 (28.33)		
Bronchitis, *n* (%)				10.63	0.001
No	109 (55.33)	79 (72.48)	30 (27.52)		
Yes	88 (44.67)	80 (90.91)	8 (9.09)		
Respiratory failure, *n* (%)				71.48	<0.001
No	176 (89.34)	157 (89.20)	19 (10.80)		
Yes	21 (10.66)	2 (9.52)	19 (90.48)		
Encephalopathy, *n* (%)				-	0.007
No	194 (98.48)	159 (81.96)	35 (18.04)		
Yes	3 (1.52)	0 (0.00)	3 (100.00)		
Paroxysmal cough, *n* (%)				5.64	0.018
No	37 (18.78)	35 (94.59)	2 (5.41)		
Yes	160 (81.22)	124 (77.50)	36 (22.50)		
Whooping, *n* (%)				4.33	0.038
No	151 (76.65)	117 (77.48)	34 (22.52)		
Yes	46 (23.35)	42 (91.30)	4 (8.70)		
Fever, *n* (%)				1.05	0.305
No	157 (79.70)	129 (82.17)	28 (17.83)		
Yes	40 (20.30)	30 (75.00)	10 (15.00)		
Vomiting, *n* (%)				0.04	0.851
No	127 (64.47)	103 (81.10)	24 (18.90)		
Yes	70 (35.53)	56 (80.00)	14 (20.00)		
Lethargy, *n* (%)				-	0.476
No	194 (98.48)	157 (80.93)	37 (19.07)		
Yes	3 (1.52)	2 (66.67)	1 (33.33)		
Cyanosis, *n* (%)				12.75	<0.001
No	148 (75.13)	128 (86.49)	20 (13.51)		
Yes	49 (24.87)	31 (63.27)	18 (36.73)		
Flushing, *n* (%)				1.50	0.220
No	46 (23.35)	40 (86.96)	6 (13.04)		
Yes	151 (76.65)	119 (78.81)	32 (21.19)		
Tachypnea, *n* (%)				25.56	<0.001
No	150 (76.14)	133 (88.67)	17 (11.33)		
Yes	47 (23.86)	26 (55.32)	21 (44.68)		

*χ*^2^, chi-square test; -, Fisher’s exact test.

**Table 3:** Length of stay of 197 hospitalized children with pertussis.

Variables	Total (*n* = 197)	Mild cases (*n* = 159)	Severe cases (*n* = 38)	*t*/*z*	*p*
Length of stay M (Q₁, Q₃)	7.00 (4.00, 12.00)	6.00 (4.00, 10.00)	16.00 (12.00, 20.00)	6.26	<0.001

In this study, the most common symptoms among hospitalized children with pertussis were pneumonia, paroxysmal cough, and flushing, observed in 120 (60.91%), 160 (81.22%), and 151 patients (76.65%), respectively. Additionally, 88 patients (44.67%) had bronchitis, 21 (10.66%) experienced respiratory failure, and three (1.52%) had encephalopathy—all of whom were admitted to the PICU. Other symptoms included whooping in 46 patients (23.35%), fever in 40 (20.3%), vomiting in 70 (35.53%), lethargy in three (1.52%), cyanosis in 49 (24.87%), and tachypnea in 47 (23.86%).

A comparison of clinical characteristics between the severe and mild cases groups revealed that the severe cases group had a longer length of stay and higher incidence rates of pneumonia, respiratory failure, encephalopathy, paroxysmal cough, cyanosis, and tachypnea than the mild cases group, with statistically significant differences (*p* < 0.05). Conversely, the severe cases group exhibited lower incidence rates of bronchitis and whooping than the mild cases group, also with statistically significant differences (*p* < 0.05).

The distribution of antibiotic treatments between the severe and mild cases groups is detailed in Table 4.

**Table 4:** Antibiotic treatments of 197 hospitalized children with pertussis.

Characteristics	Total (*n* = 197)	Mild cases (*n* = 159)	Severe cases (*n* = 38)	*χ*^2^/Fisher	*p*
Antibiotic treatment days, mean ± SD	8.67 ± 5.66	7.12 ± 4.06	16.15 ± 6.32	*t* = −7.88	<0.001
Cefuroxime, *n* (%)				*χ*^2^ = 20.10	<0.001
No	141 (71.57)	125 (78.62)	16 (42.11)		
Yes	56 (28.43)	34 (21.38)	22 (57.89)		
Amoxicillin, *n* (%)				*χ*^2^ = 4.48	0.034
No	167 (84.77)	139 (87.42)	28 (73.68)		
Yes	30 (15.23)	20 (12.58)	10 (26.32)		
Erythromycin, *n* (%)				*χ*^2^ = 0.37	0.542
No	179 (90.86)	143 (89.94)	36 (94.74)		
Yes	18 (9.14)	16 (10.06)	2 (5.26)		
Penicillin, *n* (%)				*χ*^2^ = 2.41	0.120
No	67 (34.01)	50 (31.45)	17 (44.74)		
Yes	130 (65.99)	109 (68.55)	21 (55.26)		
SMZ, *n* (%)				*χ*^2^ = 0.95	0.330
No	132 (67.01)	104 (65.41)	28 (73.68)		
Yes	65 (32.99)	55 (34.59)	10 (26.32)		

*t, t*-test, *χ*^2^: chi-square test, SD: standard deviation, SMZ: sulfamethoxazole.

Regarding antibiotic treatment, 56 patients (28.43%) received cefuroxime, 30 (15.23%) received amoxicillin, 18 (9.14%) received erythromycin, 130 (65.99%) received piperacillin, and 65 (32.99%) received sulfamethoxazole. A comparison of antibiotic treatments revealed that the severe cases group had longer durations of antibiotic treatment and higher usage rates of cephalosporins and amoxicillin than the mild cases group, with statistically significant differences (*p* < 0.05).

### 3.4 Selection of risk factors for severe pertussis

Univariate analysis identified statistically significant differences between the severe and mild cases groups in prematurity, age at onset, pertussis vaccination status, IL-5, IL-8, IL-10, TNF-*α*, IFN-α, IFN-γ, WBC count, neutrophil percentage, platelet count, hemoglobin, length of stay, pneumonia, bronchitis, respiratory failure, encephalopathy, paroxysmal cough, whooping, cyanosis, tachypnea, duration of antibiotic treatment, and usage rates of cefuroxime and amoxicillin (*p* < 0.05). These variables were found to be associated with the likelihood of hospitalized children with pertussis being admitted to the PICU. According to the study by Yao (9), factors such as length of stay and respiratory failure are characteristic of severe pertussis and are therefore not appropriate as risk or diagnostic criteria. To eliminate potential confounding factors, variables excluding length of stay and respiratory failure were included in a multivariate logistic regression model. The results indicated that cefuroxime usage rate, WBC count, and neutrophil percentage were independent risk factors for the admission of hospitalized children with pertussis to the PICU (*p* < 0.05) (Table 5).

**Table 5:** Risk factors for severe pertussis of 197 hospitalized children with pertussis on multivariate analysis.

Variables	*β*	*p*	OR	95%CI
Age	−0.01	0.806	0.99	0.90 ~ 1.09
Vaccination status				
Not vaccinated				1.00 (Reference)
Vaccinated at least one dose	1.19	0.259	3.28	0.42 ~ 25.85
Bronchitis	0.83	0.450	2.29	0.27 ~ 19.47
Paroxysmal cough	−0.90	0.559	0.41	0.02 ~ 8.28
Pneumonia	−0.45	0.743	0.64	0.04 ~ 9.46
Encephalopathy	−19.52	0.995	0.00	0.00 ~ Inf
Whooping	0.05	0.975	1.05	0.05 ~ 21.22
Cyanosis	−1.20	0.313	0.30	0.03 ~ 3.10
Tachypnea	−1.16	0.268	0.31	0.04 ~ 2.44
Cefuroxime	−2.27	0.042	0.10	0.01 ~ 0.92
Amoxicillin	0.78	0.587	2.17	0.13 ~ 35.66
White blood cell count	−0.24	0.001	0.79	0.69 ~ 0.91
Neutrophil percentage	0.40	<0.001	1.49	1.22 ~ 1.82
Platelet	0.01	0.064	1.01	1.00 ~ 1.01
Hemoglobin	−0.01	0.804	0.99	0.93 ~ 1.06

## 4 Discussion

The resurgence of pertussis has become a significant global public health threat (13). From late 2023 to early 2024, the number of reported pertussis cases in China increased rapidly, with more than 58,990 cases reported in the first quarter of 2024—10 times higher than the number reported during the same period in 2023 (14). This resurgence is attributed to various factors, including changes in pertussis transmission patterns, improved sensitivity in symptom monitoring, inadequate vaccine-acquired immunity, and increased antibiotic resistance of *B. pertussis*. As China’s demographic structure has evolved, the majority of families now have only one child, the transmission pattern of pertussis has shifted, with adolescents and adults serving as primary transmitters to infants and children (15), findings that align with this study. Infants infected with *Bordetella pertussis* are particularly prone to developing severe disease if the condition is not promptly and appropriately managed. In this study, infants with severe pertussis had significantly longer hospital stays, with the severe cases group requiring a notably longer duration of antibiotic treatment (16.15 ± 6.32 days) than the mild cases group (7.12 ± 4.06 days). This prolonged treatment imposes considerable physical and emotional burdens on the infants and their families.

All individuals are universally susceptible to pertussis, with infants exhibiting the highest incidence rates, more severe disease progression, and the greatest risk of developing severe complications (16). According to an epidemiological analysis of pertussis in China in 2022, 11,936 pertussis cases were reported among infants under 1 year old, accounting for 31.17% of all cases. Among these, infants under 1 year old represented 49.07% of hospitalized cases and 79.80% of severe cases treated in the ICU (17). Multiple studies have shown that younger infants are at a higher risk of developing severe pertussis, with age—especially under 3 months—being a significant risk factor for severe pertussis or death (18–20). In this study, severe pertussis predominantly occurred in infants under 3 months old, with 34.21% of patients in the PICU being under 3 months old, a significantly higher proportion than in the mild cases group. These findings are consistent with previous literature. Two potential reasons are as follows. First, infants under 3 months old may not have received or completed the full series of pertussis vaccinations, leading to inadequate protective immunity against pertussis (21). Second, pertussis primarily affects the respiratory mucosa, and young infants with incomplete chest development and weak cough strength are less able to clear airway secretions. Following infection, this reduced cough strength can result in airway obstruction and progression to severe pertussis.

Vaccination against pertussis is the most cost-effective method for preventing the disease and significantly reducing the risk of severe pertussis and death. Under China’s previous National Immunization Program (NIP), infants receive three doses of the diphtheria-tetanus-acellular pertussis vaccine (DTaP) at 3, 4, and 5 months of age, followed by a booster dose at 18 months. Clinical data show that the first dose of DTaP prevents 42% of pertussis-related hospitalizations, with the vaccine’s protective effect increasing with subsequent doses; completing the full four-dose series can achieve up to 100% effectiveness in preventing pertussis-related hospitalizations (22). Severe pertussis is more prevalent in infants under 3 months old, likely because of limited protection from maternal antibodies and increased susceptibility before the full-series vaccination is completed. In 2022, among infants aged 3 to 5 months in China, 49.49 and 49.28% of cases involved those who had not received or had not completed three doses of the vaccine, while infants aged 0 to 5 months had the highest proportions of hospitalizations and severe cases (17). Multiple studies have confirmed that pertussis vaccination effectively reduces hospitalization rates, alleviates clinical symptoms, and decreases the incidence of severe pertussis (23, 24). In this study, only 26.4% of patients had completed the full-series vaccination, and all infants admitted to the PICU were either unvaccinated or had not completed the full series, showing a statistically significant difference compared with the mild cases group. Our findings are consistent with recent multicenter research from Turkey, which demonstrated that infants under 3 months of age, incomplete vaccination were significantly associated with severe pertussis outcomes in PICU settings (25). Improving pertussis vaccination rates among infants, especially those under 3 months old, is critical for reducing the incidence of pertussis, lowering the risk of severe outcomes and death, and enhancing public health outcomes. Currently, China’s NIP has advanced the first dose of pertussis vaccine to 2 months of age. To better prevent severe pertussis in infants, we recommend implementing the “cocoon strategy” for pertussis vaccination—this involves advocating pertussis vaccination for pregnant women during gestation to provide protection for newborns (26).

Severe pertussis is often accompanied by various complications, including pneumonia, bronchitis, respiratory failure, and encephalopathy. In this study, factors such as pneumonia, respiratory failure, encephalopathy, paroxysmal cough, cyanosis, and tachypnea were closely associated with the admission of hospitalized children with pertussis into the PICU. Pneumonia, a common complication in severe pertussis cases, was identified as a significant risk factor for severe pertussis in infants and young children (27). In this study, 89.47% of patients in the PICU had pneumonia and 50% had respiratory failure, both showing statistically significant differences compared with the mild cases group. Thus, the presence or worsening of pneumonia in children should raise clinical suspicion for the progression of severe pertussis. *Bordetella pertussis* produces several toxins after infecting the host, with pertussis toxin (PT) being the most prominent. PT plays a key role in promoting elevated peripheral WBC counts (28), and it is widely accepted that hyperleukocytosis can lead to WBC accumulation in pulmonary arterioles, impairing pulmonary microcirculation and resulting in irreversible pulmonary hypertension, heart failure, and hypoxemia (9, 11). Multiple studies have shown a strong correlation between elevated WBC counts and accelerated disease progression, increased severity, and higher mortality in patients with pertussis (29, 30). In this study, the WBC count in the severe cases group was significantly higher than in the mild cases group, consistent with previous findings. Clinical recommendations suggest that leukapheresis (exchange transfusion) is an effective treatment for severe pertussis with hyperleukocytosis in children because it reduces peripheral WBC counts and PT levels, demonstrates good safety, and alleviates disease severity. The implementation of leukapheresis should be based on the availability of medical equipment and the expertise of healthcare providers at the treating hospital (11). Meanwhile, in mild cases, clinicians frequently detect loud coughing and potential wheezing auscultated in the lungs, leading them to be more prone to diagnosing bronchitis. However, as the disease course progresses and deteriorates, localized respiratory tract inflammation progresses to systemic complications. Consequently, clinical diagnoses shift to conditions such as respiratory failure and leukocytosis, resulting in a reduced incidence of bronchitis diagnoses. This also accounts for the higher incidence of bronchitis in the mild cases group and the typically elevated white blood cell counts in the severe cases group.

Regarding cytokines, a marked elevation of IL-8 serves as a key hallmark of severe pertussis. As a potent neutrophil chemokine, IL-8 drives the extensive recruitment and activation of neutrophils to the lungs—this directly accounts for the characteristic extreme leukocytosis and associated tissue damage observed in severe pertussis (31). Concurrently, the concurrent elevation of IL-10 carries dual implications: first, it may represent a compensatory anti-inflammatory response generated by the body to counteract excessive inflammation; second, high levels of IL-10 potently suppress macrophage and T-cell functions, resulting in immunosuppression (32). This immunosuppressive state provides a plausible explanation for the relatively low levels of TNF-α, IFN-γ, and IFN-α, which are key mediators of the Th1 immune response (33). Impairment of the Th1 response may undermine the body’s capacity to fully eliminate pathogens, consequently establishing a vicious cycle. We propose that the severity of severe pertussis in infants arises not only from excessive inflammation but, more importantly, is closely associated with this “pro-inflammatory-anti-inflammatory” response imbalance. The excessive activation of neutrophils (as characterized by IL-8), coupled with IL-10-mediated immunosuppression and impaired Th1 responses, collectively forms the critical immunological basis for the progression of pertussis to severe disease. This finding offers a novel theoretical foundation for the future development of targeted immunomodulatory therapies for severe pertussis.

Multivariate logistic regression analysis in this study identified the cefuroxime usage rate, WBC count, and neutrophil percentage as independent risk factors for severe pertussis. In the present study, the exacerbation of pertussis associated with cefuroxime use stems from multiple factors. Given the current high-level resistance of *Bordetella pertussis* to macrolide antibiotics in China, it is recommended that clinicians consider piperacillin sodium-sulbactam sodium or cefoperazone sodium-sulbactam sodium as alternative therapeutic options for infants younger than 2 months old (34). Specifically, when pediatric patients are admitted to the PICU, clinicians often tend to prioritize the administration of cefuroxime. Variability in the identification of risk factors across studies can be attributed to differences in diagnostic criteria for severe pertussis, methods of clinical information collection, intervention measures, and the autoimmune status of children (35). In summary, the resurgence of pertussis cannot be overlooked. Timely and complete pertussis vaccination, minimizing the exposure of infants—particularly those under 3 months old—to individuals with a cough, and prompt treatment of coughing infants are critical measures. Additionally, clinical indicators such as elevated WBC count and respiratory failure are significant risk factors for severe pertussis. For children with pertussis presenting with these conditions, heightened vigilance is necessary, and prompt admission to the PICU for treatment is recommended to prevent progression to severe disease.

## 5 Limitations

This study had two main limitations. First, the sample source was limited to patients treated at Children’s Hospital, Zhejiang University School of Medicine, which may not comprehensively represent infants with severe pertussis across the province. Second, nucleic acid testing for pertussis was not conducted on the family members of hospitalized children with pertussis, preventing confirmation of the family transmission pattern of the disease. Third, we adopted PICU admission as the standard for defining severe pertussis. This approach may be influenced by variations in PICU admission criteria across different medical institutions, potentially introducing a certain level of bias. Lastly, because the sample size of children who died due to severe pertussis was extremely insufficient (*n* = 4), and blood samples could not be collected from two of these children, fatal cases were excluded from the study.

## 6 Conclusion

This study retrospectively evaluated the clinical data of 197 hospitalized children with pertussis admitted to Children’s Hospital from 2019 to 2024. We found infants younger than 3 months and children who are unvaccinated or incompletely vaccinated for pertussis are more susceptible to developing severe pertussis. Common clinical features of severe pertussis include pneumonia, respiratory failure, elevated WBC count, and decreased neutrophil percentage. The aim of the study was to clarify the clinical characteristics of pertussis and the risk factors for severe pertussis, enhance the early identification of severe cases, improve the prognosis, reduce the mortality risk, and provide a reference for accurately monitoring and assessing the impact of pertussis on children’s health. Additionally, the findings aim to support future research on definitive and effective interventions.

## Data Availability

The datasets used and analysed during the current study are available from the corresponding author on reasonable request.
